# Clinical Predictors and Prevalence of Enteral Nutrition Intolerance in Acute Pancreatitis: An Updated Systematic Review and Meta-Analysis

**DOI:** 10.3390/nu17050910

**Published:** 2025-03-05

**Authors:** Wei Xiao, Yue Zeng, Lianzhong Ai, Guangqiang Wang, Yang Fu

**Affiliations:** 1 School of Health Science and Engineering, Shanghai Engineering Research Center of Food Microbiology, University of Shanghai for Science and Technology, Shanghai 200093, China; xiao18678170427@163.com (W.X.); ailianzhong1@126.com (L.A.); 2 Shanghai Key Laboratory of Pancreatic Diseases, Shanghai Jiao Tong University School of Medicine, Shanghai 201620, China; carrie_1004@sjtu.edu.cn; 3 Department of Gastroenterology, Shanghai General Hospital, Shanghai Jiao Tong University School of Medicine, Shanghai 201620, China

**Keywords:** acute pancreatitis, enteral nutrition intolerance, predictive factors, prevalence, meta-analysis

## Abstract

**Background:** Acute pancreatitis (AP) leads to severe inflammation and nutritional deficits, with 80% of severe cases experiencing critical protein loss. Timely enteral nutrition is essential for recovery. This study systematically reviews and analyzes the incidence and predictors of enteral nutrition intolerance (ENI) in AP patients. **Methods:** Web of Science, Embase, Cochrane Library, and PubMed were searched up to May 2024. Studies reporting on ENI incidence and predictors in AP patients were included based on predefined criteria. Bias was assessed using standardized tools, and meta-analyses provided summary estimates with confidence intervals. **Results:** From the 2697 screened studies, 28 involving 4853 patients met the inclusion criteria. The pooled incidence of ENI was 26%. Significant predictors included comorbid diabetes, pancreatic necrosis, elevated pre-refeeding serum lipase levels, peri-pancreatic fluid collections, and systemic inflammatory response syndrome at admission. Higher ENI rates were observed in Europe, among patients with severe acute pancreatitis (SAP), those receiving nasoenteric feeding, and in prospective study cohorts. **Conclusions:** ENI affects approximately one-quarter of AP patients and is not significantly associated with age, sex, or the cause of AP. Its incidence varies by region, disease severity, feeding method and study design. Identifying predictors, such as comorbid diabetes and pancreatic necrosis, may help clinicians reduce the risk of ENI. The limitations of this study include the heterogeneity of the included studies and inconsistent ENI diagnostic criteria.

## 1. Introduction

Acute pancreatitis (AP) is a prevalent and serious condition of the digestive system, with a global incidence rate of approximately 34 cases per 100,000 individuals [1]. Severe inflammation in AP leads to high catabolic metabolism and increased nutritional needs [2]. Approximately 80% of severe AP (SAP) patients experience substantial nutritional deficiencies, including nitrogen losses of 20 to 40 g/d [3]. Therefore, nutritional support is critical for the management of patients with AP.

Guidelines from the American Gastroenterological Association and the European Society for Clinical Nutrition and Metabolism recommend early initiation of enteral nutrition following admission [4,5,6]. Research suggests that, compared to parenteral nutrition, enteral nutrition not only provides effective nutritional support, but also enhances gastrointestinal function. It significantly reduces the incidence of infection-related complications, organ failure, the need for surgical interventions, and improves glycemic control [7,8].

Variations in patient conditions, such as underlying gastrointestinal dysmotility or elevated inflammatory responses, often induce enteral nutrition intolerance (ENI) in AP [9]. Clinically, this intolerance manifests as nausea, vomiting, abdominal distension, and pain, which can lead to prolonged hospital stays, increased healthcare utilization, and decreased quality of life [10].

The clinical significance of studying the incidence and potential risks of ENI in AP lies in its ability to improve risk stratification and optimize nutritional interventions [11]. Early identification of feeding intolerance symptoms and risk factors may allow clinicians to optimize individualized patient management, including nutritional therapy, to prevent or reduce feeding intolerance, prevent disease progression and complications, shorten hospital stays, and alleviate the economic burden associated with acute pancreatitis.

The objective of this meta-analysis was to systematically review the incidence of ENI in AP, assess the impact of confounding factors, and identify the key predictors of ENI. This study seeks to enhance the understanding of ENI, providing insights to guide clinical practice and improve nutritional support management for patients with AP.

## 2. Method

### 2.1. Registration and Protocol

The protocol was registered in the International Prospective Register of Systematic Reviews under registration number CRD42024539304, and was performed in accordance with the PRISMA 2020 checklist [12].

### 2.2. Search Strategy

Comprehensive electronic searches were conducted in databases, including Web of Science, Embase, Cochrane Library, and PubMed, covering all publications in English through to 10 May 2024. Our search strategy involved the use of Medical Subject Headings (MeSH), including “Enteral Nutrition”, “Intolerance”, and “Pancreatitis”, complemented by corresponding keywords in the titles and abstracts. The Boolean operator ‘OR’ was used to combine search terms in database queries. Additionally, manual searches of the reference lists from relevant studies were performed to identify potential omissions. The full search strategy is shown in Supplementary Table S1.

### 2.3. Inclusion and Exclusion Criteria

The inclusion criteria were as follows: studies involving adult patients (18 years or older) diagnosed with AP, using prospective, retrospective observational, or interventional designs, and assessing the incidence of ENI after enteral nutrition administration. The exclusion criteria were as follows: unpublished studies, reviews, guidelines, letters, case reports, non-English articles, conference abstracts, studies with ineligible patient populations, inappropriate intervention methods, inability to access full texts, and studies lacking viable outcome data. For studies with overlapping patient cohorts, only the study with the largest sample size was retained.

### 2.4. Literature Screening and Data Extraction

Duplicates were eliminated using Endnote X9 software. Two reviewers (Wei Xiao and Yang Fu) independently screened the titles and abstracts. Full-text articles were further assessed independently by the same reviewers, and inter-reviewer agreement was assessed using Cohen’s kappa coefficient to ensure consistency in study inclusion decisions. Any discrepancies were resolved through discussion, and in cases where consensus could not be reached, a third reviewer (Guangqiang Wang) was consulted to make the final decision. Data extraction was conducted using the Cochrane Data Extraction Form, encompassing variables such as the first author, publication year, geographic region, sample size, patient demographics (average age and sex ratio), incidence of ENI, predictors of intolerance, and outcome indicators such as tests used for ENI, severity of AP, etiology, and risk estimation using odds ratios (OR). Data from studies with multiple cohorts were included only for groups receiving enteral nutrition. If additional information was needed, the authors of eligible studies were contacted. Any disagreements were resolved by a third reviewer (Guangqiang Wang).

### 2.5. Quality Assessment

The Newcastle-Ottawa Scale (NOS) was selected to assess the methodological quality of all included studies, including randomized controlled trials (RCTs) [13]. For RCTs, only data from the enteral nutrition arms were extracted and analyzed as observational cohorts. The NOS criteria—evaluating cohort selection (0–4 stars), comparability (0–2 stars), and outcome assessment (0–3 stars)—were therefore applicable to both RCT-derived cohorts and observational studies. This approach aligned with precedents in nutritional meta-analyses and ensured uniformity in quality evaluation [14]. Any discrepancies in the assessments were resolved through discussion with the corresponding author, Yang Fu.

### 2.6. Categorization of Predictive Variables

All factors related to ENI extracted from the included studies were categorized based on the timing of measurement following the methodology of Bevan et al.: historical, at admission, and during hospitalization [14]. Factors were excluded if they relied on post-refeeding measurements (e.g., peak serum amylase levels during hospitalization), constituted study outcomes (e.g., length of hospital stay), or represented management strategies (e.g., requirements for enteral feeding or analgesics).

### 2.7. Data Synthesis and Analysis

Continuous data were analyzed for standardized mean differences (SMD) with 95% confidence intervals (CI), whereas categorical variables were assessed using odds ratios (OR) with a 95% CI to determine the effect sizes. Missing standard deviation (SD) values were calculated using the method described by Luo et al. Heterogeneity was quantified using the *I*^2^ statistic [15]. High heterogeneity (*I*^2^ > 50%) necessitated the use of a random-effects model to pool the results, whereas a fixed-effects model was used for *I*^2^ < 50%. A sensitivity analysis was conducted to identify the sources of heterogeneity. For the binary outcome data, publication bias was assessed using Harbord’s and Peter’s tests. All analyses were performed using STATA software (version 17.0; STATA, College Station, TX, USA), with statistical significance set at *p* < 0.05.

### 2.8. Subgroup and Meta-Regression Analyses

Subgroup analyses were conducted based on disease severity (mild, moderate, and severe), World Health Organization (WHO) regions (North and South America, Europe, South East Asia, and Western Pacific), and modes of feeding (oral feeding, nasogastric tube, and nasoenteric tube), study design (prospective and retrospective). The choice of disease severity as a subgroup was based on the severity of AP that can affect pancreatic exocrine function and intestinal motility, both of which are critical factors in ENI development [16]. Subgrouping by WHO regions was aimed at considering potential geographical differences in clinical practices, patient characteristics, and healthcare resources, which may influence the incidence and management of ENI [17]. The inclusion of feeding methods (oral feeding, nasogastric tube, nasoenteric tube) was based on the understanding that the route of enteral nutrition delivery can significantly affect gastrointestinal tolerance [18]. The study design subgroup was introduced to evaluate whether prospective and retrospective study designs contribute differently to the observed incidence of ENI.

Meta-regression analyses were performed to investigate potential confounding factors across all included studies, such as age (mean), sex (reference: male), etiology (reference: biliary), severity (coded as 0 = mild, 1 = moderate, 2 = severe), feeding methods (oral reference), and study design (prospective and retrospective).

## 3. Result

### 3.1. Identification of Studies

**Figure 1:**
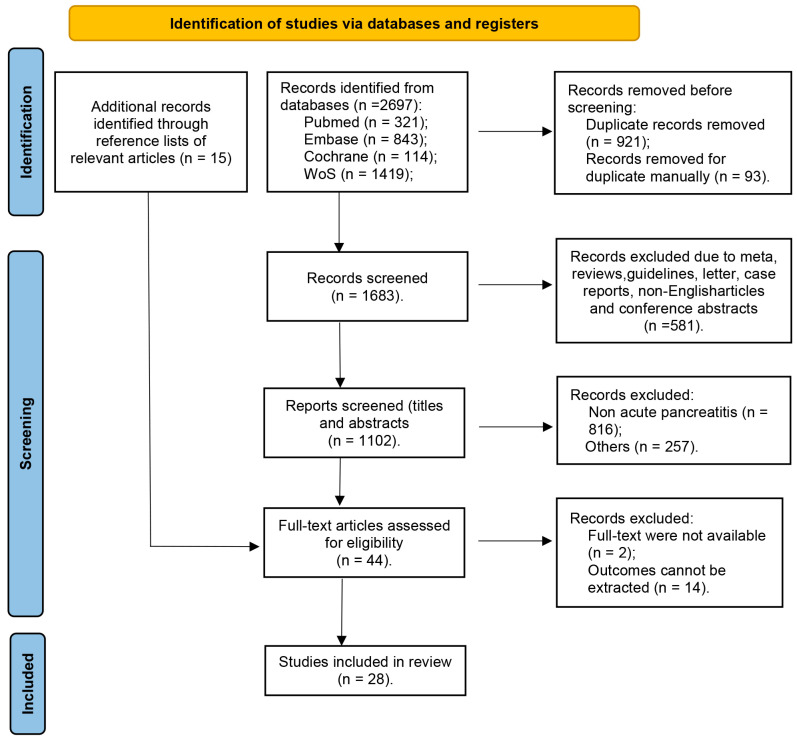
PRISMA flow diagram of the included and excluded articles.

After removing duplicates, 2697 studies were screened and 1102 were deemed eligible. Following title and abstract screenings and full-text reviews, 989 studies were excluded. Cohen’s Kappa indicated substantial agreement between the two assessors for all studies (κ = 0.78). Finally, 28 articles were included in the analysis. As shown in Figure 1, the PRISMA flow diagram provides a detailed overview of the included and excluded studies in this systematic review. For additional transparency and in adherence to PRISMA 2020 guidelines, the full PRISMA checklist is provided in Supplementary Table S2. Of these, 28 were suitable for the meta-analysis of the incidence of ENI, 27 for the meta-regression analysis, and 11 for the meta-analysis of predictive factors of ENI.

The number of records identified from each database or register searched is reported separately. The number of records excluded by human screening and automation tools is also indicated.

### 3.2. Study Characteristics

The detailed characteristics of all studies are presented in Table 1. A total of 4853 patients participated in the 28 included studies, comprising 16 interventional studies (15 randomized controlled trials [19,20,21,22,23,24,25,26,27,28,29,30,31,32,33] and 1 non-randomized trial [34]), and 12 observational studies (8 prospective [35,36,37,38,39,40,41,42] and 4 retrospective [43,44,45,46]). Information regarding the country of study, study design, severity of AP, total number of patients with AP, feeding methods, incidence of ENI, age of patients, sex, and etiology of AP is shown in Table 1.

### 3.3. Quality Assessment and Publication Bias

The quality scores are presented in Suplementary Table S3. The quality of the studies varied, with 17 of 28 studies demonstrating high methodological quality [19,21,22,23,24,27,28,30,35,36,37,38,39,40,41,43,44]. Lower scores were commonly due to potential selection bias and inadequate control of confounding factors, such as assessment timing, severity of AP, and patient age. The *p*-values obtained from Harbord’s test (*p* = 0.614) and Peter’s test (*p* = 0.458) indicated no significant evidence of publication bias in the meta-analysis data.

### 3.4. Definitions of ENI

All of the 27 included studies provided definitions of ENI, although there was significant heterogeneity among the definitions. Many studies have used a combination of clinical signs and symptoms to define ENI, which varied across studies. Supplementary Table S4 shows the detailed ENI diagnostic criteria for each study.

These definitions were categorized into three types:

1. Gastric residual volume (GRV) and/or gastrointestinal (GI) symptoms [40,43,44] and GI symptoms only [19,20,21,22,23,24,25,26,27,28,29,30,31,32,33,34,35,36,37,38,39,41,42,45,46];
2. Achievement of enteral nutrition targets [43,44];
3. Composite definitions: GRV, GI symptoms, and enteral nutrition targets [43,44].

### 3.5. Prevalence of ENI

**Figure 2:**
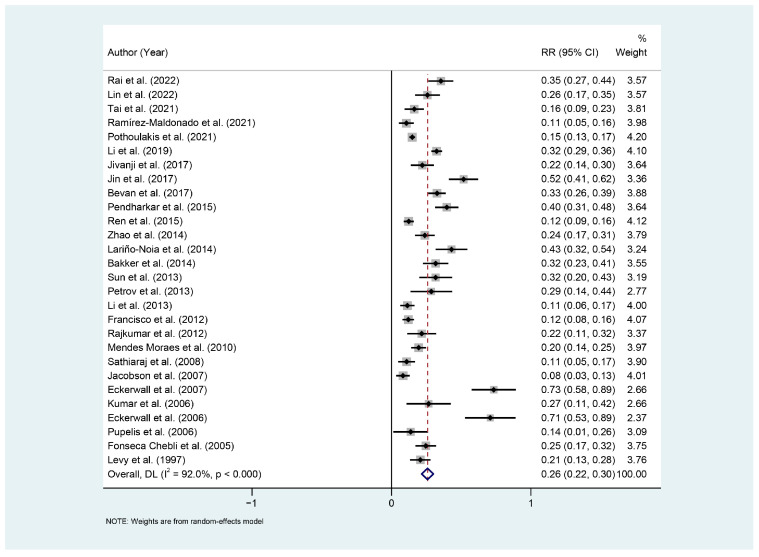
Meta-analysis of ENI incidence in patients with acute pancreatitis. Abbreviations: DL: DerSimonian and Laird; RR, rate ratio; CI, confidence interval [19,20,21,22,23,24,25,26,27,28,29,30,31,32,33,34,35,36,37,38,39,40,41,42,43,44,45,46].

The 28 studies reported the incidence of ENI in 4853 patients with AP. The pooled incidence of ENI using a random-effects model was 26% (95% CI: 0.22 to 0.30), with high statistical heterogeneity (*I*^2^ = 92.5%, *p* < 0.001) (Figure 2).

### 3.6. Heterogeneity Analysis and Sensitivity Analysis for ENI

Several pre-specified subgroup and sensitivity analyses were conducted to investigate the potential sources of heterogeneity and assess the incidence differences under varying factors and contexts.

#### 3.6.1. Subgroup Analysis for ENI

Table 2 presents the results of the subgroup analyses. A forest plot of the incidence of ENI in the subgroup analysis of patients is provided in Supplementary Figure S2.

#### 3.6.2. Meta-Regression Analyses for ENI

In the meta-regression analyses, univariate analysis indicated significant positive associations between age, sex, etiology, methodological quality, severity, and the incidence of ENI, each exhibiting a significant positive effect independently. However, when all variables were considered simultaneously in multivariate analysis, these factors did not reach statistical significance (*p* > 0.05) (Table 3). A sensitivity multivariate analysis excluding non-significant variables (feeding method, study design) was conducted. The results remained consistent, with no variables achieving statistical significance (*p* > 0.05), underscoring the stability of our conclusions (Supplementary Table S5).

#### 3.6.3. Sensitivity Analysis for ENI

The sensitivity analysis is shown in Supplementary Figure S1. The results of the sensitivity analysis indicate that the overall estimate of ENI incidence remained relatively stable after the sequential removal of each study. The sensitivity analysis confirmed the robustness of the overall ENI incidence estimate, and even with the exclusion of a single study, the analysis results did not change significantly, suggesting that the meta-analysis results are reliable.

### 3.7. Predictive Factors for ENI

Among the 28 included studies, 10 investigated 62 predictive factors for ENI [24,35,36,38,39,41,42,43,44,45]. Among these predictive factors, 9 (15%) were related to medical history, 21 (34%) were assessments or evaluations conducted at admission, and 32 (51%) were tests or evaluations conducted during hospitalization but before the introduction of enteral nutrition (Supplementary Table S6). Thirty-two predictive factors (51%) were found to be statistically significant, predominantly those assessed during hospitalization rather than at admission or based on medical history. Fourteen of the sixty-two predictors were reported by primary studies in a manner suitable for meta-analysis (Table 4). These aggregated meta-analyses revealed that comorbid conditions (diabetes), pancreatic necrosis, pre-refeeding serum lipase, (peri-)pancreatic fluid collection, systemic inflammatory response syndrome (SIRS) at admission, and uncommon etiologies were significantly associated.

## 4. Discussion

This meta-analysis integrated data from 28 cohorts (involving 4853 patients) to investigate the prevalence and predictive factors of ENI in AP. The pooled ENI prevalence was 26%, but significant heterogeneity was observed (*I*^2^ = 92.5%).

The study highlighted significant heterogeneity in the ENI definitions used across the studies evaluated. The variations in definitions, measurements of GRV, gastrointestinal symptoms, and the achievement of nutritional goals were often combined in various ways. The inconsistencies in definitions between studies underscore the urgent need for a standardized ENI definition. This heterogeneity between patient populations may partially explain the variation in incidence rates; as Blaser et al. reported, the incidence within the same population can range from 4.6% to 86.1%, depending on the definition used [47]. The ideal definition should include a comprehensive assessment of gastrointestinal symptoms, rather than a single indicator. Jenkins et al. suggested defining ENI as insufficient enteral nutrition intake (less than 80% of the target intake within 72 h of starting feeding), accompanied by one of the following symptoms: vomiting/regurgitation, bloating, or diarrhea [48]. When enteral nutrition fails to meet the recommended energy and protein intake, ENI syndrome, and gastrointestinal dysfunction should be differentiated. Additionally, potential non-enteral factors such as medications, gastrointestinal infections, and anatomical abnormalities should be considered and optimized to facilitate enteral feeding [49].

Subgroup analysis based on AP severity revealed that SAP patients had a higher incidence of ENI (38%), possibly due to systemic inflammation, impairing gut motility and permeability through dysregulated gastrointestinal hormones and neural pathways [50,51,52]. The subgroup analysis of different feeding methods showed varying incidence rates. Although recent evidence suggests that nasogastric feeding is similarly effective in reducing mortality and complications in SAP, nasoenteric feeding (32% risk) was higher than nasogastric feeding (28%) or oral feeding (23%) [53]. The geographic differences in ENI incidence further emphasize the impact of clinical practices and resource availability [54,55]. Compared to retrospective studies, prospective studies reported a lower ENI incidence. This could be due to more controlled data collection processes in prospective studies, which reduce bias. Therefore, ENI incidence is typically lower in prospective studies than in retrospective studies, as the data in retrospective studies may be subject to recall bias or incomplete reporting.

Notably, initial meta-regression linked age, sex, etiology, and severity to ENI risk, but these associations disappeared in multivariate models. This suggests collinearity between variables (e.g., SAP patients are more likely to receive tube feeding) and unmeasured confounding. Potential confounders include institutional variations in EN protocols (e.g., timing, route, formula), socioeconomic disparities in access to nutritional support, and differences in comorbidity reporting (e.g., diabetic neuropathy, chronic gastrointestinal disorders). Future studies should standardize severity stratification, control for feeding protocols, and collect detailed comorbidity data to better identify the true predictors of ENI.

The findings of this meta-analysis have significant implications for clinical practice, particularly in optimizing enteral feeding strategies for patients with AP. The identified predictors of ENI, such as comorbid diabetes, pancreatic necrosis, elevated pre-refeeding serum lipase levels, peri-pancreatic fluid collections, and systemic inflammatory response syndrome at admission, can be integrated into risk stratification models to guide clinical decision-making. This aligns with ESPEN/ACG guidelines emphasizing risk-adapted nutritional management.

For high-risk patients, such as those with diabetes, or those with pancreatic necrosis, clinicians should consider adopting the ESPEN-recommended approach: initiating enteral feeding within 24–72 h via nasogastric tube (preferred route) with continuous infusion of standard polymeric formulas. When intolerance occurs, ACG guidance advises stepwise management: (1) slowing infusion rates, (2) administering intravenous erythromycin (100–250 mg TID) for ≤3 days, and (3) transitioning to nasojejunal feeding if unresolved. Prokinetic agents should be discontinued after 72 h per consensus recommendations. These patients should be closely monitored for early signs of ENI, with EN suspension mandated if intra-abdominal pressure exceeds 20 mmHg as per ESPEN critical care guidelines. For low-risk patients, such as those without elevated pre-refeeding serum lipase levels or systemic inflammatory response syndrome at admission, the ACG-recommended strategy of initiating low-fat solid diets within 48 h of admission should be prioritized. Clinicians should encourage early oral feeding in hemodynamically stable patients post-necrosectomy, consistent with ESPEN procedural guidelines. Standard enteral nutrition protocols can be implemented using polymeric formulas via the nasogastric route if oral intake fails, with regular tolerance assessments to ensure safety and effectiveness. Future research could explore the use of innovative approaches, such as artificial intelligence, to further refine risk stratification models and improve predictive accuracy in identifying patients at high risk for ENI. AI-based tools could potentially enhance productivity in clinical decision-making and facilitate more personalized and precise nutritional interventions in patients with AP [56].

Our findings extend the seminal work of Bevan et al. (2017), the only prior meta-analysis specifically investigating feeding intolerance in AP [14]. While both studies confirm the clinical significance of peri-pancreatic collections (current OR 2.1 vs. prior OR 1.8) and elevated pre-refeeding lipase (>2.5 × ULN), our analysis extends these findings. First, our analysis encompasses all enteral feeding modalities (oral/nasoenteric/nasogastric) rather than focusing solely on oral feeding challenges, thereby capturing 58% more cases through the inclusion of 4853 patients from 28 cohorts versus 2000 patients in prior work. This broader scope revealed critical route-specific risk patterns, notably a 2.7-fold increased ENI risk with nasoenteric versus oral feeding (95%CI 1.9–3.8), a dimension absent in OFI research. We discussed and proposed clinical translations that clearly illustrate the results, providing more specific recommendations on how the identified predictive factors should guide enteral nutrition practices in patients with AP.

This study has several limitations. First, the high heterogeneity of the study designs and data (*I*^2^ = 92.5%) underscores the need for more standardized studies on the definition and assessment methods of ENI to reduce variability and provide more precise interventions. Second, while we applied the NOS uniformly to both RCTs and observational studies by treating RCT-derived cohorts as observational data (i.e., analyzing only the enteral nutrition arms), this approach inherently merges distinct study designs (RCTs and non-RCTs) without distinguishing their methodological differences. Although this allowed for consistency in quality assessment, it may obscure potential biases specific to RCTs (e.g., selection bias in intervention allocation) or observational studies (e.g., confounding by indication). To address this limitation, future research should analyze RCTs and non-RCT studies separately when assessing ENI incidence and its predictive factors. Third, the utility of predictive factors depends on the heterogeneity among the primary studies, and the unavailability of raw data limits further analysis. Although meta-analyses have been conducted, the relevant data come primarily from a limited number of studies. The analyses of comorbid conditions, pancreatic necrosis, pre-refeeding serum lipase, peri-pancreatic fluid collection, and SIRS at admission were based on a few studies; hence, their results should be interpreted with caution. Fourth, this study only included articles published in English, which may have introduced a language bias. However, considering that most of the included studies were conducted in countries where English is not the native language, this bias is unlikely to significantly affect the results.

## 5. Conclusions

In conclusion, our results indicate that nearly one-quarter of patients with AP experience ENI. Comorbid diabetes, pancreatic necrosis, elevated pre-refeeding serum lipase levels, peri-pancreatic fluid collections, and systemic inflammatory response syndrome at admission are key predictors of ENI, which should guide clinical decision-making in AP patients. High-risk patients should receive early enteral nutrition, with continuous infusion and close monitoring, while low-risk patients can benefit from early oral feeding. Further cost-effectiveness analyses and clinical trials are needed to evaluate the feasibility of using these indicators to determine optimal refeeding timing. Future research could also explore the use of innovative approaches, such as AI, to further refine risk stratification models and improve predictive accuracy in identifying patients at high risk for ENI.

## Figures and Tables

**Table 1:** Characteristics of the studies included in the systematic review.

Study	Year	Country	Study Design	Severity of AP	No. of AP Patients Included in Meta-Analysis	Feeding Methods	Incidence of ENI	Age (Mean)	Sex, No.		Etiology of AP, No.		
									Male	Female	Biliary	Alcohol	Other
Lin et al. [43]	2022	China	Retrospective observational study	68 moderate AP, 25 severe AP	93	Nasogastric tube	25.81%	40.13	63	30	36	Not stated	57
Rai et al. [19]	2022	India	Randomized controlled trial	29 severe AP, 81 moderate AP	110	Oral refeeding	35.45%	Not stated	104	6	3	101	6
Pothoulakis et al. [35]	2021	USA	Multicenter prospective observational study	909 mild AP, 262 moderate AP, 62 severe AP	1233	Oral feeding	12.98%	49.35	618	821	573	Not stated	866
Ramírez-Maldonado et al. [21]	2021	Spain	Randomized controlled trial	131 mild and moderate AP	131	Oral refeeding	10.69%	70.2	67	64	Not stated	16	115
Tai et al. [20]	2021	China	Randomized controlled trial	110 moderate AP	187	Nasogastric tube	16.36%	45.6	57	73	41	28	41
Li et al. [44]	2019	China	Retrospective observational study	568 moderate and severe	568	Nasojejunal tube	32.39%	47.46	329	239	210	147	211
Bevan et al. [38]	2017	New Zealand	Prospective observational study	Not stated	217	Oral refeeding	32.72%	49.49	114	103	30	56	131
Jin et al. [37]	2017	China	Prospective observational study	56 moderate AP, 48 severe AP	104	Nasojejunal tube	51.72%	44.68	59	28	28	16	43
Jivanji et al. [36]	2017	New Zealand	Prospective pilot study	Not stated	95	Oral refeeding	22.11%	48.87	59	36	26	22	47
Pendharkar et al. [39]	2015	New Zealand	Prospective observational study	Not stated	131	Oral refeeding	39.69%	51	62	69	61	39	31
Ren et al. [46]	2015	China	Retrospective observational study	323 mild AP	323	Oral, nasojejunal tube, nasogastric tube	12.38%	Not stated	Not stated	Not stated	Not stated	Not stated	Not stated
Bakker et al. [26]	2014	The Netherlands	Multicenter randomized controlled trial	208 severe AP	101	Nasoenteric tube	31.68%	65	89	91	115	37	53
Lariño-Noia et al. [25]	2014	Spain	Randomized controlled trial	72 mild AP	72	Oral refeeding	43.06%	59.23	33	39	40	16	16
Zhao et al. [24]	2014	China	Randomized controlled trial	101 moderate AP, 37 severe AP	138	Oral refeeding	23.91%	49.21	86	52	29	26	83
Li et al. [27]	2013	China	Randomized controlled trial	149 mild AP	149	Oral refeeding	11.41%	48.4	100	49	78	38	33
Petrov et al. [22]	2013	New Zealand	Randomized controlled trial	35 mild and moderate AP	17	Nasogastric tube	5.88%	48.82	18	17	20	8	7
Sun et al. [40]	2013	China	Prospective pilot study	60 severe AP	60	Nasojejunal feeding	31.67%	44	38	22	36	7	17
Francisco et al. [45]	2012	Spain	Retrospective observational study	232 mild AP	232	Oral refeeding	12.07%	73.37	142	110	150	25	77
Rajkumar et al. [33]	2012	India	Randomized controlled trial	60 mild AP	60	Oral refeeding	21.67%	37	55	5	5	54	1
Mendes Moraes et al. [28]	2010	Brazil	Randomized controlled trial	210 mild AP	210	Oral refeeding	19.52%	51	118	92	100	47	63
Sathiaraj et al. [29]	2008	India	Randomized controlled trial	101 mild AP	101	Oral refeeding	10.89%	38	83	18	16	51	34
Eckerwall et al. [31]	2007	Sweden	Randomized controlled trial	60 mild AP	60	Nasogastric tube	70.83%	56	13	17	Not stated	3	27
Jacobson et al. [30]	2007	USA	Randomized controlled trial	121 mild AP	121	Oral refeeding	8.26%	48.82	57	64	30	33	58
Eckerwall et al. [32]	2006	Sweden	Randomized controlled trial	50 severe AP	50	Oral refeeding	24.62%	71	10	14	Not stated	3	21
Kumar et al. [23]	2006	India	Randomized controlled trial	31 severe AP	30	Nasojejunal tube, nasogastric tube	26.67%	39.67	25	5	11	8	11
Pupelis et al. [34]	2006	Latvia	Non-randomized trial	Not stated	29	Oral refeeding	13.79%	50.66	21	8	11	18	0
Chebli et al. [41]	2005	Brazil	Prospective observational study	Not stated	130	Oral refeeding	73.33%	47	67	63	60	42	48
Levy et al. [42]	1997	France	Multicenter prospective observational study	Not stated	116	Oral refeeding	20.69%	51	74	42	54	36	26

Abbreviations: AP, acute pancreatitis. ENI, enteral nutrition intolerance.

**Table 2:** Subgroup analysis of ENI incidence in patients.

Subgroup	Numbers	RR	95% CI	*p*	*I* ^2^
Region					
North and South America	4 [28,30,35,41]	0.16	10–21	0.001	83.5
Europe	8 [21,25,26,31,32,34,42,45]	0.33	21–45	0.001	94.3
South East Asia	4 [19,23,29,42]	0.23	11–36	0.001	85.6
Western Pacific	12 [20,22,24,27,36,37,38,39,40,43,44,46]	0.27	20–34	0.001	91.9
Severity					
Mild	10 [25,27,28,29,30,31,33,45,46]	0.21	15–27	0.001	91.9
Moderately severe	3 [20,35,43]	0.16	13–21	0.742	0.01
Severe	6 [23,26,32,35,40,43]	0.38	23–54	0.001	87.9
Feeding methods					
Oral feeding	18 [19,21,24,25,27,28,29,30,31,33,34,35,36,38,39,41,42,45,46]	0.23	18–28	0.001	91.4
Nasogastric tube	5 [20,22,23,32,43]	0.28	13–43	0.001	89.6
Nasoenteric tube	5 [23,26,37,40,44]	0.32	21–42	0.001	84.9
Study design					
Prospective	23 [19,21,22,23,24,25,26,27,28,29,30,31,32,33,34,35,36,37,38,39,40,41,42]	0.27	21–23	0.001	92.4
Retrospective	5 [20,43,44,45,46]	0.20	10–29	0.001	94.5

Abbreviations: RR, rate ratio; CI, confidence interval.

**Table 3:** Results of the meta-regression analysis.

	B (95% CI)	*p*	No. of Studies Included
Univariate analyses (each variable fitted into individual models)			
Age	0.0051 (0.0039, 0.0062)	0.001	26
Sex (reference: male)	0.0009 (0.0007, 0.0011)	0.001	27
Etiology (biliary reference)	0.001 (0.001, 0.002)	0.001	24
Methodological quality	0.038 (0.029, 0.047)	0.001	28
Severty	5.7143 (4.5549, 6.8736)	0.001	16
Feeding methods (oral reference)	0.0086 (−0.0109, 0.0280)	0.307	19
Study design	−0.5009 (−2.8778, 1.8758)	0.679	28
Multivariate analyses (all variables fitted into one model)			
Age	−0.0146 (−0.224, 0.194)	0.539	26
Sex (male reference)	−0.0097 (−0.108, 0.088)	0.429	27
Aetiology (biliary reference)	0.0078 (−0.073, 0.088)	0.433	24
Methodological quality	−0.0684 (−1.056, 0.919)	0.54	28
Severty	1.5148 (−12.628, 15.658)	0.403	16
Feeding methods (oral reference)	0.001 (−0.058, 0.060)	0.86	19
Study design	−1.862 (−11.054, 7.330)	7.33	28

Abbreviations: CI, confidence interval.

**Table 4:** Meta-analyses of ENI predictors.

Classification	Sub-Classification	Predictor	Number of Studies	Pooled Estimate (95% CI)	*p*
Anamnesis	Demographics	Age	9 [30,35,36,38,39,41,42,43,44,45,46]	−0.15 (−0.47, 0.18)	0.413
		Sex	9 [30,36,39,41,42,43,45,46]	1.05 (0.94, 1.17)	0.374
	Long-term medical history	BMI	2 [36,43]	0.08 (−0.25, 0.42)	0.624
		Comorbid conditions (diabetes)	3 [36,38,46]	0.64 (0.51, 0.81)	0.001
	Symptoms before admission	Duration of symptoms before admission	6 [36,38,41,42,43,45]	0.36 (−0.07, 0.79)	0.097
Findings at admission	Clinical	APACHE II score	5 [36,38,39,43,44]	0.46 (−0.15, 1.07)	0.141
		Ranson score	4 [41,42,44,46]	0.97 (−0.35, 2.30)	0.148
		Biliary etiology	9 [35,36,38,39,41,42,43,44,45]	0.89 (0.71, 1.12)	0.321
		Alcohol etiology	7 [36,38,39,41,42,44,45]	0.92 (0.71, 1.19)	0.535
		uncommon etiologies	9 [35,36,38,39,41,42,43,44,45]	1.29 (1.12, 1.50)	0.001
		SIRS on admission	2 [35,44]	1.33 (1.22, 1.44)	0.001
Tests and outcomes during hospitalization	Clinical	Time between onset of symptoms and refeeding	2 [41,42]	−0.06 (−0.35, 0.24)	0.698
		(Peri)pancreatic collections	2 [41,45]	2.97 (1.75, 5.05)	0.001
		Pancreatic necrosis	2 [35,41]		0.001
	Laboratory	Serum amylase before refeeding	3 [41,42,46]	0.58 (−0.07, 1.23)	0.185
		Serum lipase before refeeding	3 [41,42,46]	1.29 (0.28, 2.31)	0.013

Abbreviations: CI, confidence interval; BMI, body mass index; APACHE II, Acute Physiology and Chronic Health Evaluation; SIRS, systemic inflammatory response syndrome.

## Data Availability

Data sharing is not applicable to this article as no datasets were generated or analyzed during the current study.

## Supplementary Materials

The following supporting information can be downloaded at: https://www.mdpi.com/article/10.3390/nu17050910/s1, Table S1. Literature search strategy. Table S2. PRISMA checklist. Table S3. Methodological quality scores of studies included in systematic review. Table S4. The detailed ENI diagnostic criteria for each study. Table S5. Results of meta-regression analysis. Table S6. Predictors of enteral nutrition intolerance investigated by primary studies. Figure S1. Sensitivity analysis for the meta-analysis estimates. Figure S2. Forest plots showing the incidence of ENI in subgroup analyses of patients based on different factors.

## Abbreviations

RR	rate ratio
AP	acute pancreatitis
CI	confidence interval
BMI	body mass index
APACHE II	acute physiology and chronic health evaluation
SIRS	systemic inflammatory response syndrome.
RCT	randomized controlled trial
ENI	nutrition intolerance
NOS	the Newcastle–Ottawa scale
SMD	standardized mean differences
WHO	world health organization
GRV	gastric residual volume
GI	gastrointestinal
SAP	severe AP
