# Supplementary material for: Clinical Predictors and Prevalence of Enteral Nutrition Intolerance in Acute Pancreatitis: An Updated Systematic Review and Meta-Analysis
Source: Nutrients. 2025 Mar 5;17(5):910. doi: 10.3390/nu17050910 (PMC11902221; doi:10.3390/nu17050910)
Supplement: Supplementary file 1 [file nutrients-17-00910-s001.zip › nutrients-3500681-supplementary.pdf]

Supplementary Tables

Supplementary Table S1. Literature search strategy

1.Pubmed

Search number	Query	Records
#1	"Enteral Nutrition"[Mesh]	22320
#2	((((((((((((((((((((((((Enteral Nutrition[Title/Abstract]) OR (Enteral Feeding[Title/Abstract])) OR (Force Feeding[Title/Abstract])) OR (Force Feedings[Title/Abstract])) OR (Tube Feeding[Title/Abstract])) OR (Tube Feedings[Title/Abstract])) OR (Gastric Feeding Tubes[Title/Abstract])) OR (Gastric Feeding Tube[Title/Abstract])) OR (enteric feeding[Title/Abstract])) OR (enteric nutrition[Title/Abstract])) OR (intestinal feeding[Title/Abstract])) OR (intragastric feeding[Title/Abstract])) OR (intraintestinal feeding[Title/Abstract])) OR (jejunal feeding[Title/Abstract])) OR (jejunal feedings[Title/Abstract])) OR (Oral feed[Title/Abstract])) OR (Oral refeed[Title/Abstract])) OR (Drinking[Title/Abstract])) OR (Food[Title/Abstract])) OR (Fluid[Title/Abstract])) OR (Water[Title/Abstract])) OR (Solid[Title/Abstract])) OR (Eat[Title/Abstract])) OR (Intake[Title/Abstract])) OR (Eating[Title/Abstract])	2709171
#3	(("Enteral Nutrition"[Mesh])) OR (((((((((((((((((((((((((Enteral Nutrition[Title/Abstract]) OR (Enteral Feeding[Title/Abstract])) OR (Force Feeding[Title/Abstract])) OR (Force Feedings[Title/Abstract])) OR (Tube Feeding[Title/Abstract])) OR (Tube Feedings[Title/Abstract])) OR (Gastric Feeding Tubes[Title/Abstract])) OR (Gastric Feeding Tube[Title/Abstract])) OR (enteric feeding[Title/Abstract])) OR (enteric nutrition[Title/Abstract])) OR (intestinal feeding[Title/Abstract])) OR (intragastric feeding[Title/Abstract])) OR (intraintestinal feeding[Title/Abstract])) OR (jejunal feeding[Title/Abstract])) OR (jejunal feedings[Title/Abstract])) OR (Oral feed[Title/Abstract])) OR (Oral refeed[Title/Abstract])) OR (Drinking[Title/Abstract])) OR (Food[Title/Abstract])) OR (Fluid[Title/Abstract])) OR (Water[Title/Abstract])) OR (Solid[Title/Abstract])) OR (Eat[Title/Abstract])) OR (Intake[Title/Abstract])) OR (Eating[Title/Abstract]))	2718335
#4	((((((((((((((intolerance*[Title/Abstract]) OR (intolerant[Title/Abstract])) OR (gastrointestinal intolerance[Title/Abstract])) OR (GI intolerance[Title/Abstract])) OR (gastrointestinal dysfunction[Title/Abstract])) OR (GI dysfunction[Title/Abstract])) OR (gastric emptying[Title/Abstract])) OR (gastric aspirate*[Title/Abstract])) OR (gastric residual volume*[Title/Abstract])) OR (diarrhea[Title/Abstract])) OR (diarrhoea[Title/Abstract])) OR (abdominal distension[Title/Abstract])) OR (bloat*[Title/Abstract])) OR (intestinal intolerance[Title/Abstract])) OR (intestinal ischaemia[Title/Abstract])	197354

#5	"Pancreatitis"[Mesh]	57671
#6	((((((((Pancreatitis[Title/Abstract]) OR (Pancreatitides[Title/Abstract])) OR (Pancreatic Parenchymal Edema[Title/Abstract])) OR (Pancreatic Parenchymal Edemas[Title/Abstract])) OR (Pancreatic Parenchyma with Edema[Title/Abstract])) OR (Acute Pancreatitis[Title/Abstract])) OR (Acute Pancreatitides[Title/Abstract])) OR (Peripancreatic Fat Necrosis[Title/Abstract])) OR (Peripancreatic Fat Necroses[Title/Abstract])	69194
#7	("Pancreatitis"[Mesh]) OR (((((((((Pancreatitis[Title/Abstract]) OR (Pancreatitides[Title/Abstract])) OR (Pancreatic Parenchymal Edema[Title/Abstract])) OR (Pancreatic Parenchymal Edemas[Title/Abstract])) OR (Pancreatic Parenchyma with Edema[Title/Abstract])) OR (Acute Pancreatitis[Title/Abstract])) OR (Acute Pancreatitides[Title/Abstract])) OR (Peripancreatic Fat Necrosis[Title/Abstract])) OR (Peripancreatic Fat Necroses[Title/Abstract]))	78840
#8	(((("Enteral Nutrition"[Mesh])) OR ((((((((((((((((((((((((Enteral Nutrition[Title/Abstract]) OR (Enteral Feeding[Title/Abstract])) OR (Force Feeding[Title/Abstract])) OR (Force Feedings[Title/Abstract])) OR (Tube Feeding[Title/Abstract])) OR (Tube Feedings[Title/Abstract])) OR (Gastric Feeding Tubes[Title/Abstract])) OR (Gastric Feeding Tube[Title/Abstract])) OR (enteric feeding[Title/Abstract])) OR (enteric nutrition[Title/Abstract])) OR (intestinal feeding[Title/Abstract])) OR (intragastric feeding[Title/Abstract])) OR (intraintestinal feeding[Title/Abstract])) OR (jejunal feeding[Title/Abstract])) OR (jejunal feedings[Title/Abstract])) OR (Oral feed[Title/Abstract])) OR (Oral refeed[Title/Abstract])) OR (Drinking[Title/Abstract])) OR (Food[Title/Abstract])) OR (Fluid[Title/Abstract])) OR (Water[Title/Abstract])) OR (Solid[Title/Abstract])) OR (Eat[Title/Abstract])) OR (Intake[Title/Abstract])) OR (Eating[Title/Abstract]))) AND ((((((((((((((intolerance*[Title/Abstract]) OR (intolerant[Title/Abstract])) OR (gastrointestinal intolerance[Title/Abstract])) OR (GI intolerance[Title/Abstract])) OR (gastrointestinal dysfunction[Title/Abstract])) OR (GI dysfunction[Title/Abstract])) OR (gastric emptying[Title/Abstract])) OR (gastric aspirate*[Title/Abstract])) OR (gastric residual volume*[Title/Abstract])) OR (diarrhea[Title/Abstract])) OR (diarrhoea[Title/Abstract])) OR (abdominal distension[Title/Abstract])) OR (bloat*[Title/Abstract])) OR (intestinal intolerance[Title/Abstract])) OR (intestinal ischaemia[Title/Abstract]))) AND (("Pancreatitis"[Mesh]) OR (((((((((Pancreatitis[Title/Abstract]) OR (Pancreatitides[Title/Abstract])) OR (Pancreatic Parenchymal Edema[Title/Abstract])) OR (Pancreatic Parenchymal Edemas[Title/Abstract])) OR (Pancreatic Parenchyma with	321

	Edema[Title/Abstract])) OR (Acute Pancreatitis[Title/Abstract])) OR (Acute Pancreatitides[Title/Abstract])) OR (Peripancreatic Fat Necrosis[Title/Abstract])) OR (Peripancreatic Fat Necroses[Title/Abstract])))	

2.Cochrane

Search number	Query	Records
#1	'enteric feeding'/exp	2481
#2	'enteral nutrition':ab,ti OR 'enteral feeding':ab,ti OR 'force feeding':ab,ti OR 'force feedings':ab,ti OR 'tube feeding':ab,ti OR 'tube feedings':ab,ti OR 'gastric feeding tubes':ab,ti OR 'gastric feeding tube':ab,ti OR 'enteric feeding':ab,ti OR 'enteric nutrition':ab,ti OR 'intestinal feeding':ab,ti OR 'intragastric feeding':ab,ti OR 'intraintestinal feeding':ab,ti OR 'jejunal feeding':ab,ti OR 'jejunal feedings':ab,ti OR 'oral feed':ab,ti OR 'oral refeed':ab,ti OR drinking:ab,ti OR food:ab,ti OR fluid:ab,ti OR water:ab,ti OR solid:ab,ti OR eat:ab,ti OR eating:ab,ti OR intake:ab,ti	184874
#3	#1 OR #2	185164
#4	intolerance*:ab,ti OR intolerant:ab,ti OR 'gastrointestinal intolerance':ab,ti OR 'gi intolerance':ab,ti OR 'gastrointestinal dysfunction':ab,ti OR 'gi dysfunction':ab,ti OR 'gastric emptying':ab,ti OR 'gastric aspirate*':ab,ti OR 'gastric residual volume*':ab,ti OR diarrhea:ab,ti OR diarrhoea:ab,ti OR 'abdominal distension':ab,ti OR bloat*:ab,ti OR 'intestinal intolerance':ab,ti OR 'intestinal ischaemia':ab,ti	39681
#5	'pancreatitis'/exp	1874
#6	pancreatitis:ab,ti OR pancreatitides:ab,ti OR 'pancreatic parenchymal edema':ab,ti OR 'pancreatic parenchymal edemas':ab,ti OR 'pancreatic parenchyma with edema':ab,ti OR 'acute pancreatitis':ab,ti OR 'acute pancreatitides':ab,ti OR 'peripancreatic fat necrosis':ab,ti OR 'peripancreatic fat necroses':ab,ti	4839
#7	#5 OR #6	4992
#8	#3 AND #4 AND #7	115

3.Embase

Search number	Query	Records
#1	'enteric feeding'/exp	42386
#2	'enteral nutrition':ab,ti OR 'enteral feeding':ab,ti OR 'force feeding':ab,ti OR 'force feedings':ab,ti OR 'tube feeding':ab,ti OR 'tube feedings':ab,ti OR 'gastric feeding tubes':ab,ti OR 'gastric feeding tube':ab,ti OR 'enteric feeding':ab,ti OR 'enteric nutrition':ab,ti OR 'intestinal feeding':ab,ti OR 'intragastric feeding':ab,ti OR 'intraintestinal feeding':ab,ti OR 'jejunal feeding':ab,ti OR 'jejunal feedings':ab,ti OR 'oral feed':ab,ti OR 'oral refeed':ab,ti OR drinking:ab,ti OR food:ab,ti OR fluid:ab,ti OR water:ab,ti OR solid:ab,ti OR eat:ab,ti OR eating:ab,ti OR intake:ab,ti	3286993
#3	#1 OR #2	3304006

#4	intolerance*:ab,ti OR intolerant:ab,ti OR 'gastrointestinal intolerance':ab,ti OR 'gi intolerance':ab,ti OR 'gastrointestinal dysfunction':ab,ti OR 'gi dysfunction':ab,ti OR 'gastric emptying':ab,ti OR 'gastric aspirate*':ab,ti OR 'gastric residual volume*':ab,ti OR diarrhea:ab,ti OR diarrhoea:ab,ti OR 'abdominal distension':ab,ti OR bloat*:ab,ti OR 'intestinal intolerance':ab,ti OR 'intestinal ischaemia':ab,ti	304970
#5	'pancreatitis'/exp	130784
#6	pancreatitis:ab,ti OR pancreatitides:ab,ti OR 'pancreatic parenchymal edema':ab,ti OR 'pancreatic parenchymal edemas':ab,ti OR 'pancreatic parenchyma with edema':ab,ti OR 'acute pancreatitis':ab,ti OR 'acute pancreatitides':ab,ti OR 'peripancreatic fat necrosis':ab,ti OR 'peripancreatic fat necroses':ab,ti	104948
#7	#5 OR #6	139841
#8	#3 AND #4 AND #6	843

4.Web of science

Search number	Query	Records
#1	"Enteral Nutrition (Topic) OR Enteral Feeding (Topic) OR Force Feedings (Topic) OR Tube Feeding (Topic) OR Tube Feedings (Topic) OR Gastric Feeding Tubes (Topic) OR Gastric Feeding Tube (Topic) OR enteric feeding (Topic) OR enteric nutrition (Topic) OR intestinal feeding (Topic) OR intragastric feeding (Topic) OR intraintestinal feeding (Topic) OR jejunal feeding (Topic) OR jejunal feedings (Topic) OR Oral feed (Topic) OR Oral refeed (Topic) OR Drinking (Topic) OR Food (Topic) OR Fluid (Topic) OR Water (Topic) OR Solid (Topic) OR Eat (Topic) OR Eating (Topic) OR Intake (Topic) and Preprint Citation Index (Exclude – Database)	29095073
#2	"intolerance* (Topic) OR intolerant (Topic) OR gastrointestinal intolerance (Topic) OR GI intolerance (Topic) OR gastrointestinal dysfunction (Topic) OR GI dysfunction (Topic) OR gastric emptying (Topic) OR gastric aspirate* (Topic) OR gastric residual volume* (Topic) OR diarrhea (Topic) OR diarrhoea (Topic) OR abdominal distension (Topic) OR bloat* (Topic) OR intestinal intolerance (Topic) OR intestinal ischaemia (Topic) and Preprint Citation Index (Exclude – Database)	479262
#3	"Pancreatitis (Topic) OR Pancreatitides (Topic) OR Pancreatic Parenchymal Edema (Topic) OR Pancreatic Parenchymal Edemas (Topic) OR Pancreatic Parenchyma with Edema (Topic) OR Acute Pancreatitis (Topic) OR Acute Pancreatitides (Topic) OR Peripancreatic Fat Necrosis (Topic) OR Peripancreatic Fat Necroses (Topic) and Preprint Citation Index (Exclude – Database)	137455
#4	"#1 AND #2 AND #3 and Preprint Citation Index (Exclude – Database)	1419

Supplementary Table S2. PRISMA checklist

Section and Topic	Item #	Checklist item	Location where item is reported
**TITLE**			
Title	1	Identify the report as a systematic review.	Title (pg 1)
**ABSTRACT**			
Abstract	2	See the PRISMA 2020 for Abstracts checklist.	Abstract (pg 1, line 15 - 31)
**INTRODUCTION**			
Rationale	3	Describe the rationale for the review in the context of existing knowledge.	Introduction (pg 1 - 2, line 36 - 59)
Objectives	4	Provide an explicit statement of the objective(s) or question(s) the review addresses.	Introduction (pg 2, line 60 – 63)
**METHODS**			
Eligibility criteria	5	Specify the inclusion and exclusion criteria for the review and how studies were grouped for the syntheses.	Methods (pg2, line 78 - 86, pg3-4 line 126 - 144)
Information sources	6	Specify all databases, registers, websites, organisations, reference lists and other sources searched or consulted to identify studies. Specify the date when each source was last searched or consulted.	Methods (pg2, line 69 - 77)
Search strategy	7	Present the full search strategies for all databases, registers and websites, including any filters and limits used.	Methods (pg2, line 69 - 77), Supplementary Table S1
Selection process	8	Specify the methods used to decide whether a study met the inclusion criteria of the review, including how many reviewers screened each record and each report retrieved, whether they worked independently, and if applicable, details of automation tools used in the process.	Methods (pg2 - 3, line 86 - 101)
Data collection process	9	Specify the methods used to collect data from reports, including how many reviewers collected data from each report, whether they worked independently, any processes for obtaining or confirming data from study investigators, and if applicable, details of	Methods (pg2 - 3, line 86 - 101)

Section and Topic	Item #	Checklist item	Location where item is reported
		automation tools used in the process.	
Data items	10a	List and define all outcomes for which data were sought. Specify whether all results that were compatible with each outcome domain in each study were sought (e.g. for all measures, time points, analyses), and if not, the methods used to decide which results to collect.	Methods (pg2 - 3, line 86 - 101)
	10b	List and define all other variables for which data were sought (e.g. participant and intervention characteristics, funding sources). Describe any assumptions made about any missing or unclear information.	Methods (pg2 - 3, line 86 - 101)
Study risk of bias assessment	11	Specify the methods used to assess risk of bias in the included studies, including details of the tool(s) used, how many reviewers assessed each study and whether they worked independently, and if applicable, details of automation tools used in the process.	Methods (pg3, line 102 - 110)
Effect measures	12	Specify for each outcome the effect measure(s) (e.g. risk ratio, mean difference) used in the synthesis or presentation of results.	Methods (pg3, line 86 - 101)
Synthesis methods	13a	Describe the processes used to decide which studies were eligible for each synthesis (e.g. tabulating the study intervention characteristics and comparing against the planned groups for each synthesis (item #5)).	Methods (pg3 - 4, line 126 - 142)
	13b	Describe any methods required to prepare the data for presentation or synthesis, such as handling of missing summary statistics, or data conversions.	Methods (pg3, line 115 - 124)
	13c	Describe any methods used to tabulate or visually display results of individual studies and syntheses.	Methods (pg3, line 78 - 85)
	13d	Describe any methods used to synthesize results and provide a rationale for the choice(s). If meta-analysis was performed, describe the model(s), method(s) to identify the presence and extent of statistical heterogeneity, and software package(s) used.	Methods (pg3, line 119 - 128)
	13e	Describe any methods used to explore possible causes of heterogeneity among study results (e.g. subgroup analysis, meta-regression).	Methods (pg3, line 130 - 146)
	13f	Describe any sensitivity analyses conducted to assess robustness of the synthesized results.	Methods (pg3, line 119 - 128)
Reporting bias assessment	14	Describe any methods used to assess risk of bias due to missing results in a synthesis (arising from reporting biases).	Methods (pg3, line 119 - 128)

Section and Topic	Item #	Checklist item	Location where item is reported
Certainty assessment	15	Describe any methods used to assess certainty (or confidence) in the body of evidence for an outcome.	Methods (pg3, line 102 - 105)
**RESULTS**			
Study selection	16a	Describe the results of the search and selection process, from the number of records identified in the search to the number of studies included in the review, ideally using a flow diagram.	Result (pg 4, line 149 - 157, Figure1)
	16b	Cite studies that might appear to meet the inclusion criteria, but which were excluded, and explain why they were excluded.	Result (pg 4, line 147 - 157, Figure1)
Study characteristics	17	Cite each included study and present its characteristics.	Result (pg 5, Table1)
Risk of bias in studies	18	Present assessments of risk of bias for each included study.	Result (pg 7 - 8, line 173 - 178)
Results of individual studies	19	For all outcomes, present, for each study: (a) summary statistics for each group (where appropriate) and (b) an effect estimate and its precision (e.g. confidence/credible interval), ideally using structured tables or plots.	Result (pg 4, line 191 - 193, Figure 2)
Results of syntheses	20a	For each synthesis, briefly summarise the characteristics and risk of bias among contributing studies.	Result (pg 7 - 8, line 173 - 177, Supplementary Table 2)
	20b	Present results of all statistical syntheses conducted. If meta-analysis was done, present for each the summary estimate and its precision (e.g. confidence/credible interval) and measures of statistical heterogeneity. If comparing groups, describe the direction of the effect.	Result (pg 8 - 10, line 197 – 223, Figure 2, Table 2-3)
	20c	Present results of all investigations of possible causes of heterogeneity among study results.	Result (pg 8 - 10, line 197 – 223, Table 2-3)
	20d	Present results of all sensitivity analyses conducted to assess the robustness of the synthesized results.	Result (pg 7 - 8, line 197 – 223, Supplementary Figure 1)
Reporting biases	21	Present assessments of risk of bias due to missing results (arising from reporting biases) for each synthesis assessed.	Result (pg 7 - 8, line 173 - 178)

Section and Topic	Item #	Checklist item	Location where item is reported
Certainty of evidence	22	Present assessments of certainty (or confidence) in the body of evidence for each outcome assessed.	Result (pg 7 - 8, line 173 - 178, Supplementary Table 2)
**DISCUSSION**			
Discussion	23a	Provide a general interpretation of the results in the context of other evidence.	Discussion (pg 11 - 13, line 240 - 340)
	23b	Discuss any limitations of the evidence included in the review.	Discussion (pg 11 - 13, line 240 - 340)
	23c	Discuss any limitations of the review processes used.	Discussion (pg 11 - 13, line 240 - 340)
	23d	Discuss implications of the results for practice, policy, and future research.	Discussion (pg 11 - 13, line 240 - 340)
**OTHER INFORMATION**			
Registration and protocol	24a	Provide registration information for the review, including register name and registration number, or state that the review was not registered.	Methods (pg 2, line 65 - 67)
	24b	Indicate where the review protocol can be accessed, or state that a protocol was not prepared.	https://www.crd.york.ac.uk/PROSPERO/
	24c	Describe and explain any amendments to information provided at registration or in the protocol.	NA
Support	25	Describe sources of financial or non-financial support for the review, and the role of the funders or sponsors in the review.	Pg 13, line 356 - 357
Competing interests	26	Declare any competing interests of review authors.	Pg 13, line 360
Availability of data, code and other materials	27	Report which of the following are publicly available and where they can be found: template data collection forms; data extracted from included studies; data used for all analyses; analytic code; any other materials used in the review.	Pg 13, line 358 - 359

Supplementary Table S3 Methodological quality scores of studies included in systematic review

Study	Selection (max. 4)	Comparability (max. 2)	Outcome (max. 3)	Total Score (max. 9)
Rai et al.(2022)	4	1	3	8
Lin et al.(2022)	4	1	2	7
Tai et al.(2021)	3	0	3	6
Ramírez-Maldonado et al.(2021)	4	2	2	8
Pothoulakis et al.(2021)	4	1	3	8
Li et al.(2019)	3	2	2	7
Jivanji et al.(2017)	4	1	2	7
Jin et al.(2017)	4	2	3	9
Bevan et al.(2017)	4	0	3	7
Pendharkar et al.(2015)	4	2	3	9
Sun et al.(2013)	4	1	3	8
Petrov et al.(2013)	4	0	3	7
Francisco et al.(2012)	3	2	0	5
Kumar et al.(2006)	4	0	3	7
Zhao et al.(2014)	4	0	3	7
Ren et al.(2015)	3	2	1	6
Lariño-Noia et al.(2014)	3	0	2	5
Bakker et al.(2014)	4	0	2	6
Li et al.(2013)	4	0	3	7
Mendes Moraes et al.(2010)	4	0	3	7
Sathiaraj et al.(2008)	4	0	1	5
Jacobson et al.(2007)	4	0	3	7
Eckerwall et al.(2007)	4	0	2	6
Eckerwall et al.(2006)	3	0	0	3
Chebli et al.(2005)	4	2	1	7
Levy et al.(1997)	3	2	1	6
Rajkumar et al.(2012)	2	0	2	4
Pupelis et al.(2006)	3	0	0	3

Supplementary Table S4 The detailed ENI diagnostic criteria for each study

Study	ENI diagnostic criteria
Rai et al.(2022)	Abdominal pain, nausea, vomiting, abdominal distention
Lin et al.(2022)	Large gastric residual volumes (grv>500 ml/6h), abdominal pain, vomiting due to delayed gastric emptying, failed to reach the target (70% of the estimated target) within 72 hours of en initiation
Tai et al.(2021)	Diarrhea, abdominal distention, abdominal pain
Ramírez-Maldonado et al.(2021)	Abdominal pain, nausea, vomiting, ap relapse
Pothoulakis et al.(2021)	Abdominal pain, vomiting
Li et al.(2019)	Recurrent pain, distension, nausea followed by vomiting, failed to reach the target (20 kcal/kg bw/day)
Jivanji et al.(2017)	Recurrent pain
Jin et al.(2017)	Vomiting, abdominal distention, abdominal discomfort, diarrhea
Bevan et al.(2017)	Recurrent pain
Pendharkar et al.(2015)	Pain relapse, nausea, vomiting, pain medication escalation
Sun et al.(2013)	High gastric residual volume (200 ml), repeated nausea, vomiting, abdominal pain or distension, diarrhea, aspiration
Petrov et al.(2013)	Pain relapse, nausea, vomiting
Francisco et al.(2012)	The appearance of pain, nausea, vomiting
Kumar et al.(2006)	Recurrence of pain, diarrhea
Zhao et al.(2014)	Recurrence of pain, abdominal distension, abdominal pain
Ren et al.(2015)	Abdominal pain, vomiting, gastric bleeding
Lariño-Noia et al.(2014)	Abdominal pain, nausea, vomiting
Bakker et al.(2014)	Nausea,vomiting, aspiration, lleus, diarrhea
Li et al.(2013)	Abdominal pain, transitional abdominal distension
Mendes Moraes et al.(2010)	Abdominal pain
Sathiaraj et al.(2008)	Pain, nausea, vomiting
Jacobson et al.(2007)	Pain, nausea, vomiting
Eckerwall et al.(2007)	Pain, nausea, vomiting, gripes, diarrhea
Eckerwall et al.(2006)	Abdominal pain

Chebli et al.(2005)	Relapse of abdominal pain
Levy et al.(1997)	Pain relapse
Rajkumar et al.(2012)	Recurrence of pain, vomiting, diarrhea
Pupelis et al.(2006)	Bloating, epigastric pain, liquid stool
Rai et al.(2022)	Abdominal pain, nausea, vomiting, abdominal distention

Abbreviations: ENI, enteral nutrition intolerance

Supplementary Table S5 Results of meta-regression analysis

	B (95% CI)	p	No. of studies included
Multivariate analyses (all variables fitted into one model)			
Age	-0.132 (-0.63, 0.37)	0.421	26
Sex (male reference)	-0.076 (-0.268, 0.115)	0.247	27
Aetiology (biliary reference)	0.026 (-0.207, 0.26)	0.672	24
Methodological quality	-0.25 (-0.59, 0.09)	0.453	28
Severty	11.0189 (-16.81, 38.85)	0.231	16

Abbreviations: CI, confidence interval.

Supplementary Table S6. Predictors of enteral nutrition intolerance investigated by primary studies

Classification	Sub-classification	Predictor	Study	P value
Anamnesis	Demographics	Age	Chebli et al. 2005	NS
			Francisco et al. 2012	NS
			Jacobson et al. 2007	NS
			Levy et al. 1997	NS
			Lin et al. 2022	NS
			Pendharkar et al. 2015	NS
			Pothoulakis et al. 2021	<0.02
			Li et al. 2019	NS
			Jivanji et al. 2017	NS
			Bevan et al. 2017	NS
			Ren et al. 2015	NS
		Sex	Chebli et al. 2005	NS
			Francisco et al. 2012	NS
			Jacobson et al. 2007	NS
			Levy et al. 1997	NS
			Pendharkar et al. 2015	NS
			Lin et al. 2022	NS
			Pothoulakis et al. 2021	<0.01
			Li et al. 2019	NS
			Jivanji et al. 2017	NS
			Ren et al. 2015	NS
	Long-term medical history	BMI	Jacobson et al. 2007	NS
			Ren et al. 2015	NS
			Lin et al. 2022	NS
			Pothoulakis et al. 2021	NS
			Jivanji et al. 2017	NS
		Comorbid conditions	Francisco et al. 2012	NS
			Ren et al. 2015	NS
			Jivanji et al. 2017	NS
			Bevan et al. 2017	NS
		Previous pancreatitis	Jacobson et al. 2007	NS
			Ren et al. 2015	<0.05
		Active alcohol use	Pothoulakis et al. 2021	<0.05
		Active smoking	Pothoulakis et al. 2021	<0.05
	Symptoms before admission	Duration of symptoms before admission	Chebli et al. 2005	<0.01
			Francisco et al. 2012	<0.02
			Levy et al. 1997	NS
			Lin et al. 2022	NS

			Jivanji et al. 2017	NS
			Bevan et al. 2017	NS
			Ren et al. 2015	<0.05
		Time between onset of symptoms and stopping oral intake	Levy et al. 1997	NS
Findings at admission	Clinical	AP severity score	Chebli et al. 2005	NS
			Lin et al. 2022	<0.01
			Francisco et al. 2012	NS
			Jacobson et al. 2007	NS
			Levy et al. 1997	<0.02
			Pothoulakis et al. 2021	NS
			Pendharkar et al. 2015	NS
			Ren et al. 2015	NS
		Aetiology	Chebli et al. 2005	NS
			Lin et al. 2022	NS
			Francisco et al. 2012	NS
			Jacobson et al. 2007	
			Levy et al. 1997	NS
			Pendharkar et al. 2015	<0.01
			Ren et al. 2015	NS
			Pothoulakis et al. 2021	<0.01
			Li et al. 2019	NS
			Jivanji et al. 2017	NS
			Bevan et al. 2017	<0.02
		Gallstones or bile duct stones	Francisco et al. 2012	<0.01
			Ren et al. 2015	NS
		Temperature	Francisco et al. 2012	NS
			Jacobson et al. 2007	NS
		Systolic blood pressure	Francisco et al. 2012	NS
		Heart rate	Francisco et al. 2012	NS
		Presenting symptom	Ren et al. 2015	NS
		Respiratory failure	Lin et al. 2022	<0.05
		Acute Kidney Injury	Lin et al. 2022	<0.02
		Shock	Lin et al. 2022	NS
		SIRS on admission	Pothoulakis et al. 2021	<0.01
			Li et al. 2019	0.05
		Ranson score	Li et al. 2019	NS
			Ren et al. 2015	NS
			Chebli et al. 2005	NS
			Levy et al. 1997	NS
		APACHE II score on day of refeeding	Jacobson et al. 2007	NS

			Lin et al. 2022	<0.05
			Li et al. 2019	NS
			Jivanji et al. 2017	NS
			Bevan et al. 2017	NS
	Laboratory	Serum amylase	Chebli et al. 2005 Francisco et al. 2012	NS
			Francisco et al. 2012	NS
		Serum lipase	Chebli et al. 2005	NS
			Jacobson et al. 2007	NS
			Pothoulakis et al. 2021	<0.02
		CRP	Li et al. 2019	NS
		Blood glucose	Jivanji et al. 2017	NS
			Francisco et al. 2012	NS
		Creatinine	Francisco et al. 2012	NS
		Urea	Pothoulakis et al. 2021	<0.01
			Francisco et al. 2012	NS
		Haematocrit	Francisco et al. 2012	<0.05
			Pothoulakis et al. 2021	NS
		Bilirubin	Francisco et al. 2012	NS
		Aspartate aminotransferase	Francisco et al. 2012	NS
Tests and outcomes during hospitalisation	Clinical	Time between onset of symptoms and refeeding	Chebli et al. 2005	NS
			Jacobson et al. 2007	NS
			Levy et al. 1997	NS
			Li et al. 2019	<0.01
		Time between end of pain and starting refeeding	Levy et al. 1997	NS
		Abdominal pain on day of refeeding	Jacobson et al. 2007	<0.05
		Total duration of pain	Levy et al. 1997	<0.02
		Fasting time	Francisco et al. 2012	<0.01
		Pleural effusion	Francisco et al. 2012	NS
		Enlarged Wirsung duct	Levy et al. 1997	NS
		Vomiting	Levy et al. 1997	NS
		Pancreatic necrosis	Chebli et al. 2005	NS
			Pothoulakis et al. 2021	<0.01
		(Peri)pancreatic collections	Chebli et al. 2005	<0.01
			Francisco et al. 2012	NS
		Abdominal collections	Levy et al. 1997	NS
		Ileus	Chebli et al. 2005	NS
		Initial full diet	Francisco et al. 2012	<0.01
			Ren et al. 2015	<0.05

	Immediate oral refeeding	Ren et al. 2015	<0.001
	Pancreatic enzyme supplementation	Ren et al. 2015	NS
	SIRS at 48 h	Pothoulakis et al. 2021	0.01
	GI function	Li et al. 2019	<0.01
	Pancreatic infection	Li et al. 2019	<0.01
	IV hydration first 48 hours	Francisco et al. 2012	NS
Laboratory	Serum amylase before refeeding	Chebli et al. 2005	<0.01
		Levy et al. 1997	NS
		Ren et al. 2015	NS
	Serum lipase before refeeding	Chebli et al. 2005	<0.01
		Levy et al. 1997	<0.03
		Ren et al. 2015	<0.01
	CRP before refeeding	Ren et al. 2015	NS
	Day 4 CRP	Chebli et al. 2005	<0.01
	WBC count first 48 hours	Francisco et al. 2012	NS
		Jacobson et al. 2007	NS
	Blood glucose concentration before refeeding	Jivanji et al. 2017	NS
	Glucose variability	Jivanji et al. 2017	NS
	Mean amplitude of glycemic excursions	Jivanji et al. 2017	NS
	Mean blood glucose concentration	Jivanji et al. 2017	NS
Instrumental investigations	CT in first 48 hours	Francisco et al. 2012	<0.02
		Levy et al. 1997	NS
	ERCP in first 48 hours	Francisco et al. 2012	<0.02
		Levy et al. 1997	NS
	MRCP performed	Francisco et al. 2012	NS

Abbreviations: NS, non-significant; BMI, body mass index; AP, acute pancreatitis; CRP, c-reactive protein; APACHE, acute physiology and chronic health evaluation; IV, intravenous; WBC, white blood cell; CT, computed tomography; ERCP, endoscopic retrograde cholangiopancreatography; MRCP, magnetic resonance cholangiopancreatography.

Supplementary Figures

Supplementary Figure S1. Sensitivity analysis for the meta-analysis estimates.

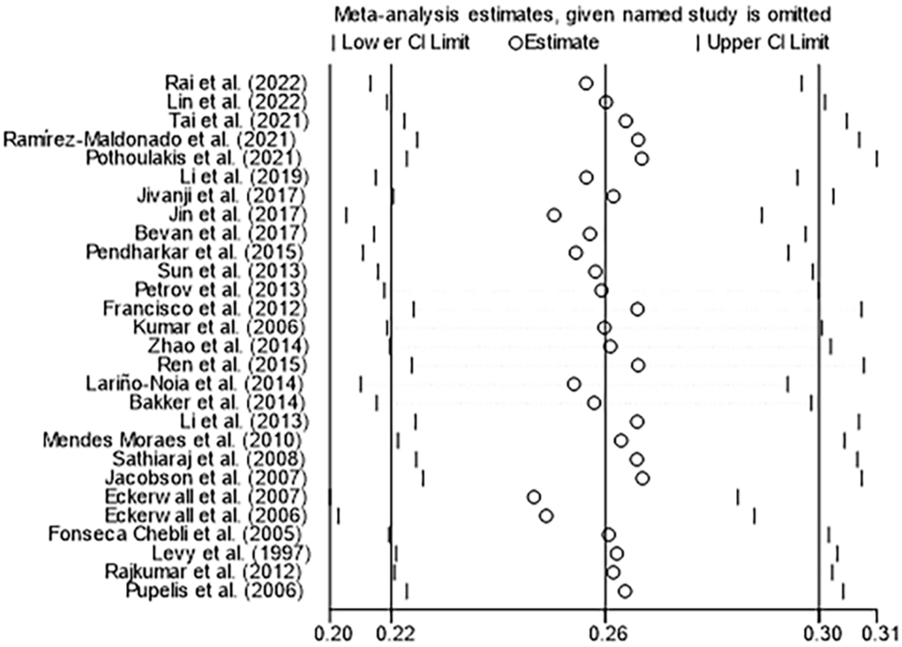

Abbreviations: CI, confidence interval.

Supplementary Figure S2 Forest plots showing the incidence of ENI in subgroup analyses of patients based on different factors

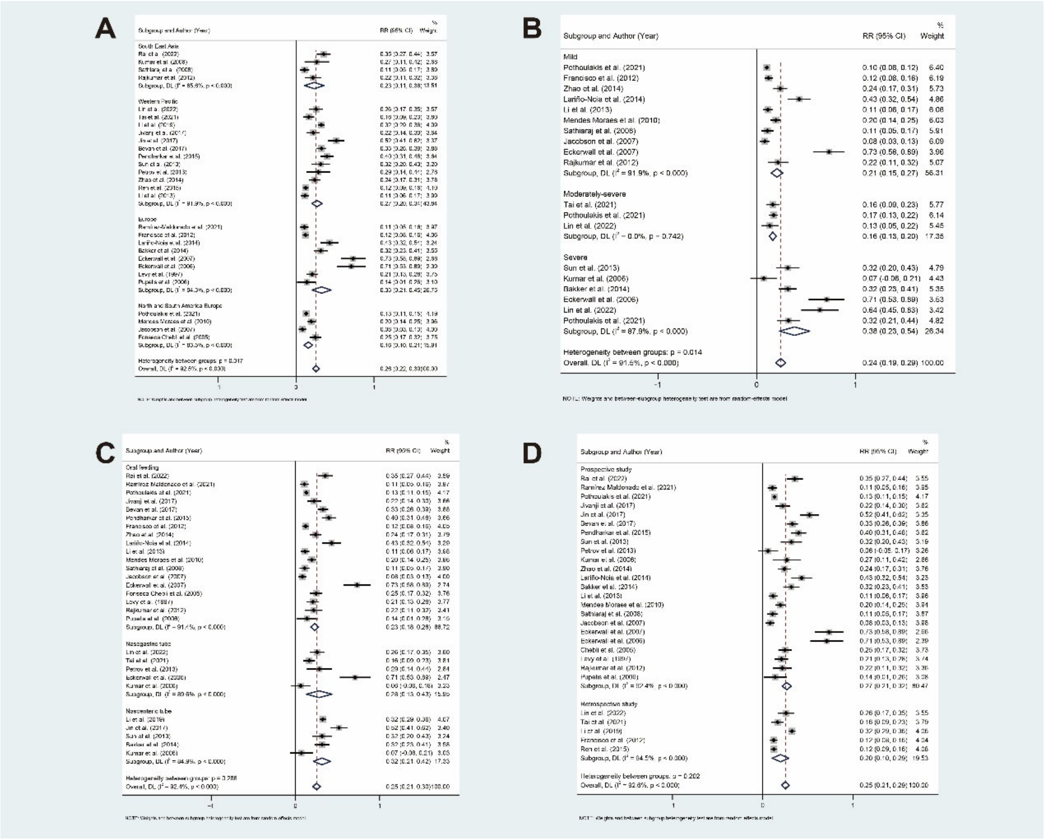

(A) Subgroup analysis based on the regional. (B) Subgroup analysis based on the severity of acute pancreatitis (AP). (C) Subgroup analysis based on feeding methods. (D) Subgroup analysis based on study design.

Abbreviations: DL: DerSimonian and Laird, RR, rate ratio; CI, confidence interval.
